# Electrophoretic mobility confirms reassortment bias among geographic isolates of segmented RNA phages

**DOI:** 10.1186/1471-2148-13-206

**Published:** 2013-09-24

**Authors:** Samuel L Díaz-Muñoz, Olivier Tenaillon, Daniel Goldhill, Kristen Brao, Paul E Turner, Lin Chao

**Affiliations:** 1 Section of Ecology, Behavior and Evolution, University of California San Diego, 9500 Gilman Drive, Muir Building 3155, La Jolla, CA 92093-0116, USA; 2 Inserm U722, Faculté de Médecine Xavier Bichat, 16, rue Henri Huchard, Paris 75018, France; 3 Department of Ecology and Evolutionary Biology, Yale University, New Haven, CT 06520, USA

**Keywords:** Sex, Population structure, Hybridization, Cooperation, Experimental evolution, Ecology

## Abstract

**Background:**

Sex presents evolutionary costs and benefits, leading to the expectation that the amount of genetic exchange should vary in conditions with contrasting cost-benefit equations. Like eukaryotes, viruses also engage in sex, but the rate of genetic exchange is often assumed to be a relatively invariant property of a particular virus. However, the rates of genetic exchange can vary within one type of virus according to geography, as highlighted by phylogeographic studies of cystoviruses. Here we merge environmental microbiology with experimental evolution to examine sex in a diverse set of cystoviruses, consisting of the bacteriophage ϕ6 and its relatives. To quantify reassortment we manipulated – by experimental evolution – electrophoretic mobility of intact virus particles for use as a phenotypic marker to estimate genetic exchange.

**Results:**

We generated descendants of ϕ6 that exhibited fast and slow mobility during gel electrophoresis. We identified mutations associated with slow and fast phenotypes using whole genome sequencing and used crosses to establish the production of hybrids of intermediate mobility. We documented natural variation in electrophoretic mobility among environmental isolates of cystoviruses and used crosses against a common fast mobility ϕ6 strain to monitor the production of hybrids with intermediate mobility, thus estimating the amount of genetic exchange. Cystoviruses from different geographic locations have very different reassortment rates when measured against ϕ6, with viruses isolated from California showing higher reassortment rates than those from the Northeastern US.

**Conclusions:**

The results confirm that cystoviruses from different geographic locations have remarkably different reassortment rates –despite similar genome structure and replication mechanisms– and that these differences are in large part due to sexual reproduction. This suggests that particular viruses may indeed exhibit diverse sexual behavior, but wide geographic sampling, across varying environmental conditions may be necessary to characterize the full repertoire. Variation in reassortment rates can assist in the delineation of viral populations and is likely to provide insight into important viral evolutionary dynamics including the rate of coinfection, virulence, and host range shifts. Electrophoretic mobility may be an indicator of important determinants of fitness and the techniques herein can be applied to the study of other viruses.

## Background

Sex – defined as the exchange of genetic material between individuals during reproduction – has evolutionary benefits and costs. Asexual populations maintain co-adapted gene complexes [1], but sex allows purging of deleterious mutations [2] and allows beneficial mutations to be fixed in the same background [3]. The costs and benefits of sex are likely to vary according to ecological conditions, yet in viruses –long models for the study of sex – variation in genetic exchange across different environments is understudied. In viruses, genetic exchange takes the form of recombination [4], when information from two template strands is incorporated to a daughter strand, or reassortment [5], in which viruses with segmented genomes exchange chromosomes.

Sex can have profound effects on viral evolutionary dynamics including host range emergence, escape from immunity, increased virulence, among others. However, systematic information on the rates of genetic exchange among different viruses is scarce [6]. In particular, reassortment seems to be somewhat restricted in some viruses [7] whereas it is very commonplace in others – e.g. influenza viruses [8], orbiviruses [9], cystoviruses [10]. Restricted reassortment is often explained due to selection against reassortants and/or coadptation of genomic segments [11]. However, in other viruses selection clearly acts in favor of reassortants, such as cases of host emergence in influenza virus [12] and hantavirus [13].

Reported variation in reassortment among viruses is likely influenced by the dramatic differences in genome structure, expression and replication [6]. A less appreciated cause is geographic variation in reassortment rates *within* a particular virus group [14]. Geography can influence reassortment rates in several ways. First, environmental effects on viral abundance may dictate opportunities for coinfection, influencing the probability that different genotypes infect the same host. In turn, viruses may evolve to be more or less prone to reassort according to the benefits and costs dictated by the environment [15]. Thus, while it is likely that different viruses vary in their reassortment rates, an investigation of different geographical areas is key to ascertaining the range of variation within each particular virus.

Here we investigate the rates of reassortment in a diverse set of cystoviruses, most of which were isolated from the environment, primarily from natural settings. Cystoviruses are lytic, lipid enveloped, dsRNA viruses with tripartite genomes of ~13 kbp composed of a small, medium, and large segment. The first member of the *Cystoviridae*, ϕ6, was isolated from *Pseudomonas*-infected bean straw by Vidaver [16] and remained its monotypic representative until additional cystoviruses were isolated from plants in agricultural [17] and natural settings [10,14]. The lipid envelope of ϕ6 is similar in fatty acid composition to the cytoplasmic membrane of *Pseudomonas syringae* pv *phaseolicola*[18], which has traditionally been used to propagate ϕ6 and other cystoviruses in the lab. Although cystoviruses are able to infect other bacteria [17], the host range in natural settings awaits investigation and they are assumed to primarily infect pseudomonads.

Two recent studies concluded that reassortment rates in naturally isolated cystoviruses differed by geographic region. Phylogenetic analyses of partial genome sequences of cystoviruses isolated from plants in California [10] and the Northeastern United States [14] suggested differences in population genetic structure including reassortment. The patterns seen in phylogenetic analyses may be the signature of historical processes or, instead, reflect contemporary gene flow [19]. Experimental tests of reassortment can illuminate this question by testing the propensity of strains to create hybrid (reassortant) progeny. Here we combine environmental microbiology with experimental evolution to examine reassortment in a diverse set of cystoviruses.

We used ϕ6, a common lab strain, as a reference point to assay other cystoviruses, to estimate their propensity to hybridize (reassort). We employed experimental evolution to generate variability in electrophoretic mobility for use as a novel phenotypic marker for examining hybridization rates. Electrophoresis has been used previously to investigate intact virus particles’ properties [20], including hybridization [21]. However, we manipulated electrophoretic mobility in order to create a selectable phenotypic marker for ϕ6 that can be used in very low cost assays to provide an estimate of the level of genetic exchange between strains.

Here we: 1) present the results of experimental evolution to select for slow and fast electrophoretic mobility in bacteriophage ϕ6; 2) sequence the genomes of selected strains to reveal sequence changes associated with mobility phenotypes; 3) demonstrate the utility of mobility differences to detect hybridization; 4) apply the technique to determine whether and at what rate a collection of environmental isolates of cystoviruses can hybridize with ϕ6; and 5) examine differences in the hybridization rate according to geography.

## Methods

### Study strains, culture conditions and crosses

Bacteriophage strains used in experiments are listed in Table 1. These included ϕ6, originally isolated by Vidaver [16] and obtained from the American Type Culture Collection (ATCC no. 21781-B1), and a collection of cystoviruses of known phylogenetic relatedness isolated more recently in California [10] and the Northeastern US [14]. We also used phages isolated from agricultural samples of unknown geographic origin [17]. The bacterial host for all strains was *Pseudomonas syringae* pv *phaseolicola* (*Pp*) (strain HB10Y, ATCC no. 21781). Growth, plating, dilution, and incubation were conducted at 25°C in LC media [22] at a pH of 7.5, using previously described procedures [15]. Crosses between strains were single burst lysates as previously described [23], but at a multiplicity of infection (MOI) of 10.

**Table 1:** **Strains of the*Cystoviridae*used in this study, including geographic origin, the proportion of hybrids generated when crossed with ϕ6-Fast1 (as determined by normal mixture models), and the mean electrophoretic mobility (relative to xylene cyanol) of each strain**

**Strain**	**Proportion of hybrids in cross with ϕ6-Fast1**	**Mean electrophoretic mobility relative to xylene cyanol (Rf ± SD)**	**Geographic origin**	**Reference**
CA/KW051	0.204	0.60 ± 0.11	California	[10]
CA/KW065a	0.319	0.74 ± 0.22	California	[10]
CA/KW065d	0.391	0.68 ± 0.08	California	[10]
CA/KW066c	0.089	0.69 ± 0.11	California	[10]
CA/KW068	0.204	0.82 ± 0.17	California	[10]
CA/KW073	0.100	0.66 ± 0.21	California	[10]
KRI289	0.046	0.36 ± 0.03	Rhode Island	[14]
KRI300	0.000	0.46 ± 0.13	Rhode Island	[14]
KRI301	0.000	0.71 ± 0.12	Rhode Island	[14]
KRI319	0.000	0.37 ± 0.10	Rhode Island	[14]
NCT94	0.160	0.33 ± 0.07	Connecticut	[14]
ϕ7	0.355	0.49 ± 0.13	Unknown	[17]
ϕ9	0.000	0.49 ± 0.18	Unknown	[17]
ϕ10	0.230	0.40 ± 0.14	Unknown	[17]
ϕH2	0.050	1.00 ± 0.23	Lab-Evolved from ϕ6	[24]
ϕ6-Slow1	0.822	0.80 ± 0.43	Lab-Evolved from ϕ6	This Study
ϕ6-Fast1		1.46 ± 0.40	Lab-Evolved from ϕ6	This Study
ϕ6-Slow2		0.76 ± 0.40	Lab-Evolved from ϕ6	This Study
ϕ6-Fast2		1.19 ± 0.34	Lab-Evolved from ϕ6	This Study
ϕ6 (ATCC 21781-B1)		0.94 ± 0.33	Nebraska	[16]

### Quantification of electrophoretic mobility

We sought to use electrophoretic mobility of whole, intact virus particles as a phenotypic marker. We took the following steps to measure electrophoretic mobility of strains: We ran approximately 20 μL of lysate (at concentrations of 10^7^-10^11^ particles/mL) with 5 μL of blue/orange loading dye at 70 V at 4°C in a 0.8% agarose/TAE gel. We rinsed the gel with distilled water and sampled (poked) two times with an 8-channel pipette outfitted with tips. The sampling points were offset in order to create 16 sampling points along the length of the 9.5 cm gel tray. After sampling, we immediately inserted the pipette tips into a microtiter plate with LC media and serially diluted. We plated serial dilutions from the microtiter plate on a lawn of *Pp* to obtain a virus concentration for each sampling point. We used sampling points as reference points to assign Rf values (Retention factor, i.e. relative mobility), relative to the migration of xylene cyanol, in order to control for differential migration during separate gel runs. We tabulated and graphed the concentration of phage at each sampling point (transformed to Rf values) in order to obtain a distribution of the abundance of phage particles throughout the length of the gel lane.

### Selection for fast and slow electrophoretic mobility

We ran a high titer lysate of ϕ6 (ATCC no. 21781-B1) on an agarose gel, as described above, until xylene cyanol reached a pre-determined point on the gel (corresponding to sampling point 6). We determined mobility as described above. In order to select for fast and slow moving phage particles, we excised a section of the gel corresponding to either tail of the mobility distribution (calculated from the previous gel run). We placed this gel section in LC media, serially diluted, and plated on a *Pp* lawn to recover ~10^4^ phage. The top agar layer with plated phages was filtered and purified to create a lysate, which formed the basis for the following round of selection. We iterated this selection process in two independent replicates to yield 4 strains hereby named: ϕ6-Fast1, ϕ6-Slow1, ϕ6-Fast2, ϕ6-Slow2.

### Genome sequencing of strains with fast and slow electrophoretic mobility

To identify the genetic changes in the strains subjected to experimental evolution of electrophoretic mobility, we sequenced whole genomes of ϕ6-Fast1, ϕ6-Slow1, ϕ6-Fast2, ϕ6-Slow2, as well as the ancestor, ϕ6. We extracted genomic material from high titer lysates representing a population sample of each strain, using a QiaAMP Viral RNA extraction kit (Qiagen Inc., Valencia, CA). We reverse transcribed extractions by RT-PCR with Superscript polymerase and random hexamer primers (Invitrogen, Carlsbad, CA). We purified PCR amplicons of the genome using gel extraction (QIAquick, Qiagen Inc., Valencia, CA) and sequenced using standard Sanger sequencing methods. Sequences are available in Genbank under the following accession numbers: KF615858 - KF615869.

### Crosses of strains of different electrophoretic mobility

In order to confirm that ϕ6 strains selected for slow and fast electrophoretic mobility would be useful in studies of reassortment, we crossed slow and fast phage lysates in a single burst experiment [23] at a multiplicity of infection (MOI) of 10 to determine whether intermediate mobility phenotypes were produced. We self-crossed each of the parental strains in a single burst experiment with the same MOI (10) as a control. We compared the mean mobilities for control parental self-crosses and the slow/fast cross with ANOVA and Tukey HSD tests.

The gel assay uses a phenotypic trait to estimate genetic reassortment. We took additional steps to ensure that intermediate phenotypes corresponded to hybrid genotypes, and thus, that the phenotypic measurements would reasonably estimate genetic exchange. Single burst lysates are composed of the assembled viruses resulting from the coinfection of parental strains. It is possible that during coinfection, strains may exchange proteins with other strain(s) that do not correspond to their genotypes. This is known as phenotypic mixing. When viral lysate has been prepared after a single burst, these assembled viruses have not been given the chance to express their own genotypes, because they have not infected a bacterial cell. Thus, it is in principle possible that some viral proteins do not reflect the genotype of an individual virion. Since single burst lysates were run directly on the gel (in absence of bacteria), it is possible to have a mismatch between phenotypic and genotypic mixing (reassortment). To exclude this possibility, we plated single burst lysates resulting from a cross of ϕ6-Fast1 and ϕ6-Slow1 as single plaques, harvested into a new lysate, and then subjected to the same gel assay. The only difference between these lysates, is that the latter has allowed individual virions to reproduce clonally in cells to allow expression of their genotypes. We compared the results of these gel runs using two sample statistical tests. Additionally, we isolated 10 clones from the single burst lysate and determined the mobility of each of those, to determine whether intermediate phenotypes were recovered from clones.

### Hybridization rate estimation

The single burst lysate resulting from a cross contains viral progeny with some combination of the six parental segments. When run on a gel, progeny phage with both parental and hybrid segment combinations are represented in the distribution generated by sampling the gel. We crossed all cystoviruses isolated from the environment against a standard: the population lysate of a ϕ6 strain, ϕ6-Fast1, with manipulated electrophoretic mobility from selection experiments described above. Using a known standard allowed quantification of differences in hybridization rate across environmental strains. We used self-crossing of parental phages (i.e. environmental strain and ϕ6-Fast1) in a single burst experiment with the same MOI (10), as a control. Additionally, we used ϕH2 [24] in a test cross. This strain was evolved under a high coinfection regime and acts as a “cheater”, i.e. it outcompetes other coinfected viruses such that the vast majority of progeny viruses will bear the parental ϕH2 segments, to the exclusion of hybrids and the other parent. The known strategy of this strain served as further corroboration of hybridization estimation methods.

We quantified electrophoretic mobility as described above, except that we diluted by serially poking the pipette tips directly on a plate with a *Pp* lawn. This modification was repeatable and increased throughput in the measurement of electrophoretic mobility. We generated mobility distributions using data from three independent gel runs of the same single burst lysate of each cross.

We used finite Gaussian mixture models to determine the proportion of parental and hybrid progeny represented in the single burst lysate. We used the package mixtools[25] in the R statistical programming environment [26] to estimate the proportions of mixture components. Because the distribution of parentals was known from control crosses, we input the mean migration value for parentals as prior information to fit the component mixture. The normal models in mixtools use an expectation maximization algorithm (EM) to find maximum likelihood estimates of parameters of incomplete observations. The EM algorithm has a stochastic element, so we run models with varying inputs to verify results. In particular, we tested an unsupervised classification (i.e. no prior information of means or variances of components) with three components to verify if the assigned components coincided with the parental controls and an intermediate hybrid. We used the package mclust 3[27] – which also uses an EM algorithm, but uses the Bayesian Information Criterion to determine the optimal number of mixture components – as a second method to verify consistency of results. We considered the proportion assigned to the intermediate component to be the rate of hybridization for the environmental strain being tested.

Crosses between strains may sometimes fail to yield hybrid progeny. Since hybrids may be present at low frequencies and may not be detected by our method, we adopted conservative criteria to conclude that the single-burst lysate was a two-component mixture, i.e. a hybridization rate of zero. We determined that a lysate from a cross was a two component mixture if: a) the proportion of a component in a mixture was lower than 0.01 or the means of mixture components differed by less than 0.1, b) the component distribution means were not significantly different (*t*-test), and c) likelihood ratio bootstrapping (mixtools) and Bayesian Information Criterion (mclust 3) of mixture models with different numbers of components determined a two component mixture was more likely.

Again, to ensure that the mixed phenotypes assayed on the gel corresponded to mixed genotypes, we plated clonal progeny from three of the crosses to allow individual virions to express their genotypes. We subjected these pooled progeny to the same gel assay and repeated hybridization rate estimates from the mixture models. Additionally the means of the hybrid component predicted from the two alternate assays were compared using two-sample tests.

## Results

### Selection for fast and slow electrophoretic mobility

**Figure 1:**
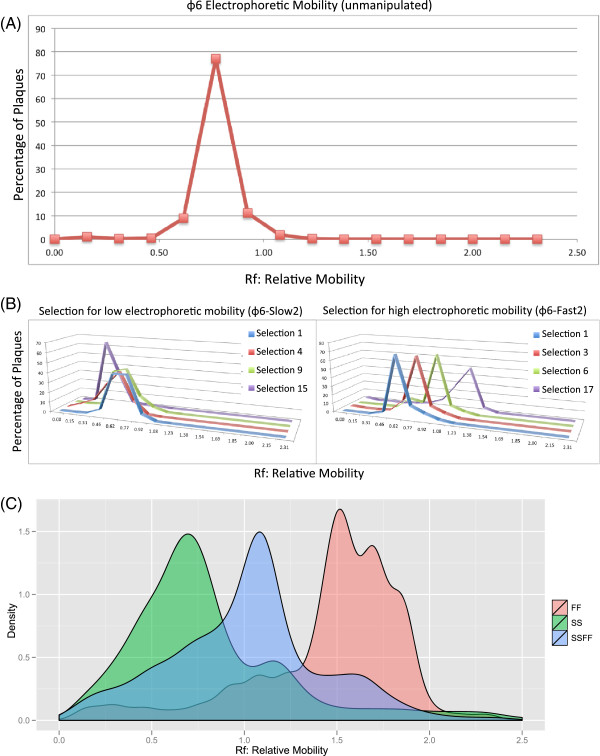
**Selection on ϕ6 for fast and slow electrophoretic mobility. (A)** Migration rate on agarose electrophoresis gel of ϕ6 virus lysate without manipulation. Rf is the migration of xylene cyanol dye relative to sampling points. **(B)** The progression of migration rate during the electrophoretic mobility selective regime. Four representative strains of ϕ6 derived strains along the slow (left panel) or fast (right panel) selection regime are shown. These represent ancestors of ϕ6-Slow2 and ϕ6-Fast2, respectively. Each line of a different color represents a strain from a given round of selection, i.e. passage number. **(C)** Density plot of relative mobility of ϕ6-Slow1 (SS), ϕ6-Fast1 (FF), and the single burst lysate of their cross at a multiplicity of infection of 10 (SSFF).

We sought to generate strains that exhibited slower or faster electrophoretic mobility as compared to unmanipulated ϕ6. To achieve this goal, we iterated the selection process for mobility for 27, 25, 18, and 18 rounds of selection, respectively for ϕ6-Fast1, ϕ6-Slow1, ϕ6-Fast2 and ϕ6-Slow2. Figure 1B illustrates the progression of the selection process over successive rounds of selection (i.e. passage generations). The experiment yielded strains that differed significantly in mobility from unmanipulated ϕ6 (ANOVA: *F* = 207.3, *p* < 0.001, Figure 1C). Selection for fast mobility yielded greater difference from unmanipulated ϕ6, compared to slow-mobility selection (Tukey HSD: ϕ6 vs. ϕ6-Fast1 = -0.521, 95% CI: -0.601 -0.441; ϕ6 vs. ϕ6-Slow1 = 0.135, 95% CI: 0.055-0.215).

### Genome sequencing of strains with fast and slow electrophoretic mobility

**Figure 2:**
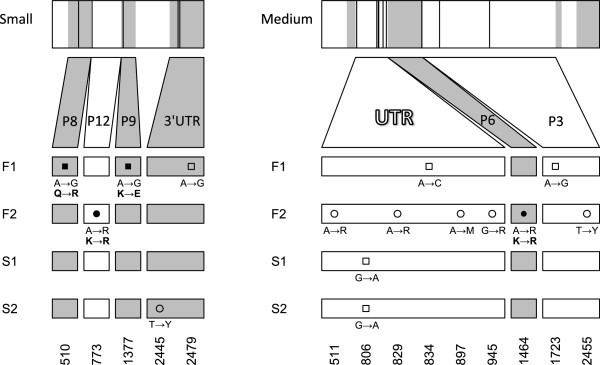
**Results of whole genome sequencing of ϕ6 strains selected for fast and slow mobility on an agarose gel.** There were no mutations on the large segment. Squares are fixed mutation, circles are polymorphisms, filled is non-synonymous and open is synonymous. Amino acid changes are listed in bold below corresponding sequence changes. Shaded areas (in gray) indicate the physical location of genetic changes within the genome. The numbers indicate the positions of nucleotide changes in the genome.

Genome sequencing revealed several genetic changes in ϕ6 strains selected for fast and slow mobility (Figure 2). All mutations occurred in the small or medium segments: no polymorphisms were detected in the large segment. Both ϕ6-Slow1 and ϕ6-Slow2 had the same synonymous polymorphism (G > A) in an untranslated region (UTR) of the medium segment preceding proteins P6 and P3. The only other mutation in the strains selected for slow mobility was in ϕ6-Slow2, a synonymous polymorphism on the 3' UTR region of the small segment. The ϕ6-Fast strains did not share any polymorphisms or mutations, however there were regions of the genome that exhibited changes in both strains: the UTR region in the medium segment and the P3 gene both had synonymous polymorphisms. Point mutations leading to amino acid changes were found in genes P8 and P9 in ϕ6-Fast1 and genes P12 and P6 in ϕ6-Fast2. All strains subjected to selection had one or more mutations in the medium segment UTR adjacent to proteins P6 and P3.

### Creation of hybrid with intermediate electrophoretic mobility

**Figure 3:**
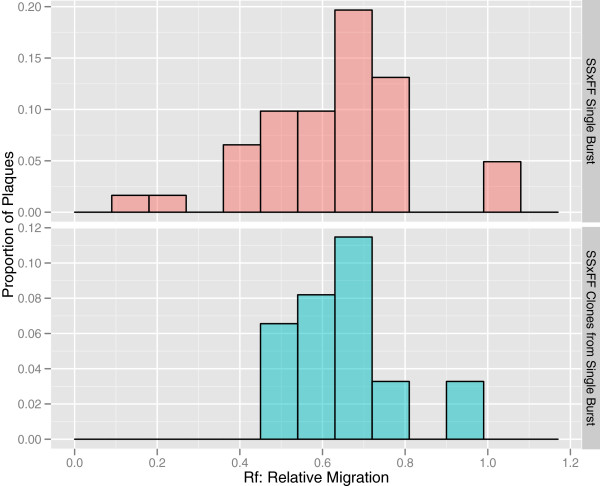
Distributions of relative mobility on electrophoresis gel of single burst lysates resulting from a cross of ϕ6-Fast1 and ϕ6-Slow1 (top) and a second lysate where the single burst lysate was plated as single plaques (i.e. ~200 plaques plates without overlap) and harvested into a new lysate (bottom).

**Figure 4:**
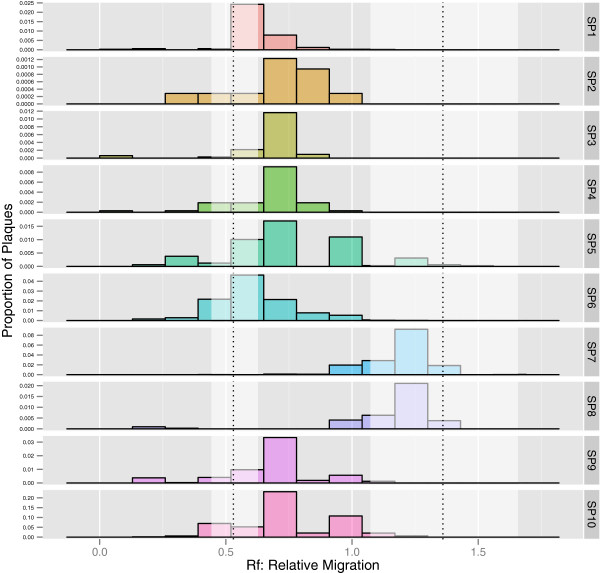
**Mobility of 10 clones (single plaques) isolated from a single burst lysate.** The dotted lines indicate mean mobility for ϕ6-Fast1 and ϕ6-Slow1 and the white shading around them indicates 1 standard deviation from those means.

To validate the utility of electrophoretic mobility as a marker to detect hybrid viruses, we crossed two strains selected for fast and slow mobility (ϕ6-Fast1 and ϕ6-Slow1) at a high MOI (10). The distribution of the single burst lysate created from the cross of ϕ6-Slow and ϕ6-Fast falls in between parentals with intermediate mean mobility (ANOVA: *F* = 165.4, *p* < 0.001; Figure 1B). To ensure that this intermediate phenotype corresponded to a hybrid genotype, we conducted two additional experiments to exclude phenotypic mixing of viruses (as opposed to genetic exchange) as the source of this phenotype. First, lysates harvested from clones derived from the single burst lysate (to allow clones to express their genotypes) resulted in distributions that were not significantly different from the single burst lysate (W = 397, p-value = 0.8442, Figure 3), suggesting genotypic mixing occurred and hybrids had been generated. Additionally, we ran ten single plaques derived from the cross of ϕ6-Fast1 and ϕ6-Slow1 individually on the gel to quantify mobility. Mobility of these clones spanned the range between ϕ6-Fast1 and ϕ6-Slow1 (Figure 4), including intermediate hybrids.

### Hybridization estimates for environmental strains and relationship to geography

**Figure 5:**
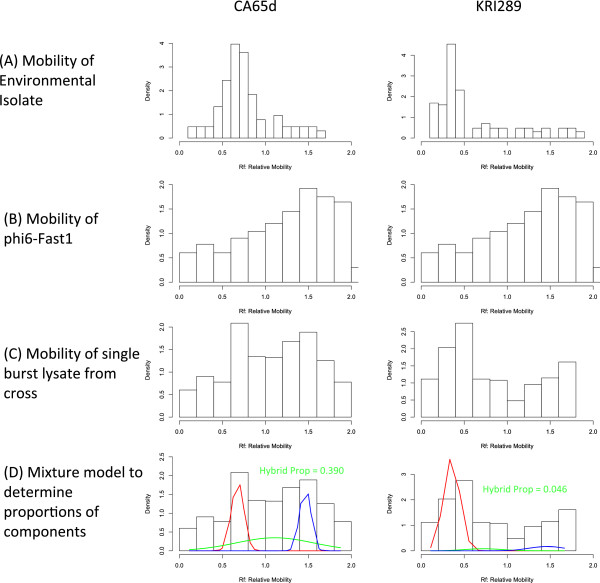
**Illustration of steps taken to estimate hybridization rate, using finite mixed models. (A)** The mobility of a given environmental isolate was characterized, by running a control self cross on the gel. **(B)** The same procedure was used to determine the mobility of ϕ6-Fast1. **(C)** The mobility of the cross (single burst) lysate was quantified. **(D)** A mixture model was parameterized with the mobility of the environmental strain (from **A**), in blue, and the ϕ6-Fast1 reference strain (from **B**), in red, and the model determined the proportion of a third component, in green, which was the estimate of hybridization for that strain.

In order to generate baseline estimates of hybridization rate for the collection of environmental samples, we selected a strain to serve as a standard for all crosses. Selection on fast mobility yielded greater difference from unmanipulated ϕ6 compared to slow mobility. Therefore, ϕ6-Fast1 was chosen as the standard for crosses with the collection of environmental strains. Representative graphs illustrating results from mixture analyses are presented in Figure 5.

**Figure 6:**
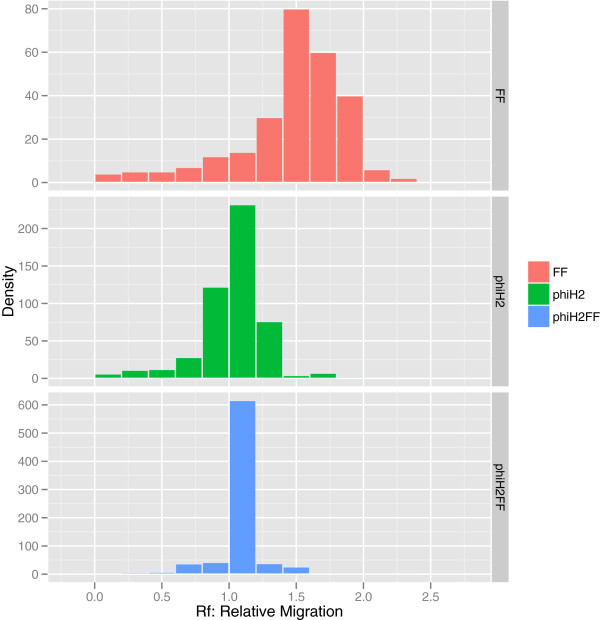
Histogram of electrophoretic mobility of ϕ6-Fast1 (FF), ϕΗ2 cheater strain (phiH2), and the single burst lysate of their cross at a multiplicity of infection of 10 (phiH2FF).

For further validation of our method for hybridization estimation, we conducted a test cross with ϕH2, which has a “cheating” strategy during coinfection. Test crosses with ϕH2 and ϕ6-Fast1 confirmed the known reassortment strategy of ϕH2: the ϕH2 parental component – as calculated by mixtools and mclust 3 – dominated the single burst lysate (proportion > 0.90) at the expense of hybrid and ϕ6-Fast1 phenotypes (Figure 6).

To confirm that mixed phenotypes assayed on the gel corresponded to mixed genotypes, we repeated the same mixture models for a subset of samples (CA65a × ϕ6-Fast1, KRI300 × ϕ6-Fast1, ϕΗ2 × ϕ6-Fast1), but with a lysate derived from single plaques of the single burst lysate. These samples were chosen to capture the different hybridization patterns we observed, including samples that yielded no hybrids. The hybridization rate estimate obtained from these samples was not different from the single burst lysates and captured the range of hybridization dynamics (Table 2).

**Table 2:** A comparison of results from mixture models run on data derived from single burst assays (SBL) and a lysate derived from single plaques harvested from the SBL, allowing an opportunity for clones to express their genotypes without other strains in the cell

**Cross**	**CA65a × ϕ6-Fast1,**		**KRI300 × ϕ6-Fast1**		**ϕΗ2 × ϕ6-Fast1**	
	**SBL**	**SBL-SP’s**	**SBL**	**SBL-SP’s**	**SBL**	**SBL-SP’s**
Number of components	3	3	2	2	1	1
Estimated hybridization rate	0.34	0.36	0	0*	0	0*
Mean ± SD Rf of largest mixture component	0.997 ± 0.401	1.210 ± 0.046	0.463 ± 0.142	0.458 ± 0.335	0.995 ± 0.188	0.995 ± 0.465
*t*-test for largest mixture component	t = 3.15, df = 137, p = 0.002 95% CI: 0.0790 0.347		t = -0.11, df = 227, p = 0.916 95% CI: -0.113 0.101		t = 0, df = 169, p = 1 95% CI: -0.116 0.116	

*KRI300 and PT309 fail to produce hybrids with ϕ6-Fast1 in different ways: the KRI300 cross recovers both parental phenotypes and no intermediates, whereas ϕΗ2 recovers only its own parental phenotype at the expense of the ϕ6-Fast1 and hybrid phenotypes, confirming its advantage in coinfection.

**Figure 7:**
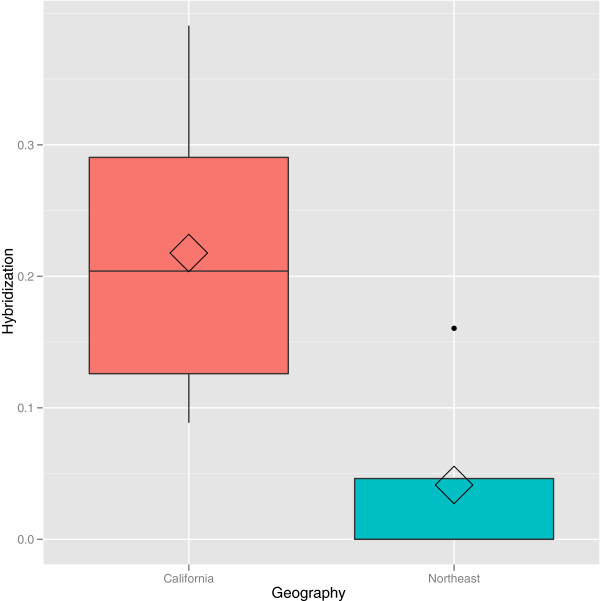
**Boxplot of hybridization rate according to geographic origin of cystoviruses.** Diamond shows mean values for hybridization rate.

To explicitly test the influence of geography on reassortment rates, we compared hybridization estimates from samples of known geographic origin and found that California strains had higher hybridization rates than those isolated in the Northeasten United States (Wilcoxon rank sum test: W = 28, HL∆ = 0.166, HL∆ 95% CI = 0.043-0.344, p = 0.021, Figure 7). Three out of five strains from the Northeastern US (and none from California) showed no detectable hybridization with ϕ6, meeting aforementioned criteria to select a two-component mixture as the most appropriate model. Estimates of hybridization rate and the geographic origin of environmental isolates are presented in Table 1.

## Discussion

### Selection for and measurement of electrophoretic mobility

Electrophoretic mobility was useful as a phenotypic marker for estimating hybridization, as shown previously for ϕX174 [21]. Here we adapted this approach, but selected directly for electrophoretic mobility in ϕ6 in order to have a standard to compare to other cystoviruses isolated from the environment. Thus, environmentally sampled viruses could be crossed with minimal manipulation and lysates could be assayed after single burst experiments. The main advantage of this method is as a low-cost assay to estimate reassortment. Electrophoretic mobility is a selectable marker that can be of use in future studies in the *Cystoviridae*, or may be applied to other viruses.

Genome sequencing of fast and slow-moving ϕ6 strains identified genetic changes that occurred during the experimental evolution regime. These sequence changes may affect phenotypic traits that underlie electrophoretic mobility. While a variety of factors can cause differences in electrophoretic mobility on an agarose gel, charge, mass, and shape are likely candidates for component traits underlying mobility. The genetic mutations observed suggest potential phenotypes. First, all strains exhibited some genetic change in the same medium segment untranslated region (Figure 2). This region is believed to be involved in differential expression of P3 and P6 envelope proteins, which would change the shape and total charge of different strains. Additionally, there was an amino acid substitution in protein P9 of the ϕ6-Fast1 strain that changes a positively charged amino acid into a negatively charged one. We observed more mutations in the fast moving strains, consistent with our observation from the phenotypic assays that selection for fast mobility progressed more rapidly. Future studies will attempt to understand the component traits affecting mobility, as well as their genetic bases in order to determine the action of selection on each component and its impact on fitness.

We note that this study documents natural variability in the electrophoretic mobility of phages isolated from the environment. While electrophoretic mobility was used purely as a convenient phenotypic marker for this study, the traits underlying electrophoretic mobility in viruses are very likely of evolutionary significance. These traits can be major determinants of fitness for microbes and viruses. A variety of microbes change charge to avoid host immune defenses [28,29]. Size can also influence viral fitness as in HIV, where variation in particle size can make structural approaches to vaccine design cumbersome [30], providing HIV with an escape from antiviral attack.

### Geographic differences in reassortment

Phylogenetic analyses have suggested differences in reassortment among cystoviruses in different locations [10,14], finding that California samples showed higher rates of genetic exchange than Northeastern US samples. Yet this phylogenetic observation could derive from many different scenarios. For instance, population demography, the lack of opportunities for exchange due to alternate hosts in the wild, or differences in reassortment efficiency are all possible factors. An essential first step to determine whether reasortment could shape the genetic structure of wild phage, is to experimentally cross them to determine if hybrid phage are produced. With the present experiment, we take this first step and show that reassortment is possible and definitely contributing to the observed patterns: The patterns of phylogenetic structure in cystoviruses [14] match reassortment efficiency in the lab, at least when measured against ϕ6, suggesting that sex is a contemporary process actively shaping phylogenetic structure. However, it is important to note that this study only examined reassortment patterns against a single reference phage (ϕ6-Fast1) and these patterns may vary with other reference phage. ϕ6 is a well-known strain that was separate from the sample of natural isolates, and thus represented an appropriate, albeit initial, yardstick to estimate reassortment in cystoviruses. Nonetheless, this study establishes that reassortment can play a role in the genetic structure of phage and that these patterns are consistent with previously reported patterns of genetic exchange estimated by phylogenetics [14], thus paving the way for future work examining reassortment dynamics in detail for a variety of cystoviruses.

Evidence from laboratory experiments with ϕ6 suggest that the rates of reassortment are influenced by the coinfection regime in which the virus evolved [15]. If this relationship holds in natural populations, the results of this paper may provide insight into the rates of coinfection at different locations. We first confirmed, through an independent method, the known strategy of a “cheater” virus [24]. Interestingly, some of the Northeastern US samples qualitatively appeared to follow the cheater’s pattern, with increased representation of the self-parental phenotype at the expense of hybrid and the other parental phenotype (Figure 5: KRI289). The Northeastern US samples had a lower reassortment rate, which could be indicative of a lower realized MOI in nature, whereas lab studies suggest the cheating phenotype arises in high MOI conditions. However, fitness assays comparing coinfection and single infections are necessary to confirm if indeed some environmental isolates follow a cheating strategy.

Insights into reassortment and rates of coinfection have important implications for virus population structure in nature, which remains largely unexplored [31-33]. Uncovering the geographic variation in reassortment rates among viral populations may not only allow the delimitation of viral populations, but also provide a deeper understanding of viral evolutionary dynamics. For instance, on one hand, targeted surveillance of areas with high levels of reassortment may enhance the detection of pathogens emerging through reassortment. On the other hand, viruses under high coinfection regimes frequently face increased intrahost competition [15], sometimes leading to the creation of defective interfering particles that happen to be attractive candidates for attenuated vaccines [34]. Hence, “prospecting” in areas of high reassortment may yield viable vaccine candidates for a variety of viruses.

## Conclusions

In sum, electrophoretic mobility is a useful, selectable phenotypic marker for studies of virus sex and could itself be an indicator of important determinants of virus fitness. The results confirm that cystoviruses from different geographic locations have remarkably different reassortment rates –despite similar genome structure and replication mechanisms– and that these differences are in large part due to sexual reproduction. This suggests that particular viruses may indeed exhibit diverse sexual behavior, but wide geographic sampling, across varying environmental conditions may be necessary to characterize the full repertoire, as the scale at which viral populations occur may be vastly different than for macro-organisms. Variation in reassortment rates suggests varied cost-benefit equations for sex, having implications for local evolutionary dynamics and a suite of virus characteristics including rates of coinfection, virulence and host range shifts.

### Data availability

The data set supporting the results of this article is available in the Dryad repository, DOI: 10.5061/dryad.6qh25, and genome sequences have been deposited in GenBank under the following accession numbers: KF615858 - KF615869.

## Competing interests

The authors declare no competing interests.

## Authors' contributions

SLDM and LC conceived and designed the study. OT and PET assisted in study design and analysis. SLDM and OT conducted experimental evolution of electrophoretic mobility. DG, KB, and PET conducted genome sequencing and analysis. SLDM conducted electrophoresis mobility assays, statistical analyses, and drafted the manuscript. SLDM, OT, DG, KB, PET, and LC participated in manuscript preparation and approved the final version. All authors read and approved the final manuscript.
